# Feasibility and Role of Cardiac Magnetic Resonance in Intensive and Acute Cardiovascular Care

**DOI:** 10.3390/jcm14041112

**Published:** 2025-02-09

**Authors:** Isabella Leo, Stefano Figliozzi, Jessica Ielapi, Federico Sicilia, Daniele Torella, Santo Dellegrottaglie, Anna Baritussio, Chiara Bucciarelli-Ducci

**Affiliations:** 1 Royal Brompton and Harefield Hospitals, Guys and St Thomas NHS Foundation Trust, London SW3 6NP, UK; isabella.leo@unicz.it; 2 Department of Experimental and Clinical Medicine, Magna Graecia University, 88100 Catanzaro, Italyfederico.sicilia@studenti.unicz.it (F.S.); dtorella@unicz.it (D.T.); 3 IRCCS Humanitas Research Hospital, Via Manzoni 56, 20089 Rozzano, Italy; 4 Department of Advanced Biomedical Sciences, University of Naples Federico II, Via Pansini, 80131 Napoli, Italy; 5 School of Biomedical Engineering & Imaging Sciences, King’s College London, London WC2R 2LS, UK; 6 Advanced Cardiovascular Imaging Unit, Clinica Villa dei Fiori, 80011 Acerra, Italy; 7 Department of Cardiac Thoracic Vascular Sciences and Public Health, Padua University Hospital, 35128 Padua, Italy

**Keywords:** cardiac magnetic resonance, tissue characterization, acute heart failure, cardiomyopathies

## Abstract

Cardiac magnetic resonance (CMR) is established as a key imaging modality in a wide range of cardiovascular diseases and has an emerging diagnostic and prognostic role in selected patients presenting acutely. Recent technical advancements have improved the versatility of this imaging technique, which has become quicker and more detailed in both functional and tissue characterization assessments. Information derived from this test has the potential to change clinical management, guide therapeutic decisions, and provide risk stratification. This review aims to highlight the evolving diagnostic and prognostic role of CMR in this setting, whilst also providing practical guidance on which patients can benefit the most from CMR and which information can be derived from this test that will impact clinical management.

## 1. Introduction

In the setting of acute cardiovascular disease, the imaging diagnostic work-up often starts with transthoracic echocardiography, which is quick, widely available, low-cost, and radiation-free [1]. However, this modality is also highly dependent on an acoustic window that can be suboptimal in ventilated and non-cooperative patients admitted to the intensive care units (ICUs). Cardiac computed tomography (CT) provides the rapid, high-resolution imaging of cardiac and vascular structures; however, its use requires a careful risk–benefit assessment due to radiation exposure and the potential risk of contrast-induced nephropathy, particularly in vulnerable patients [2].

In patients presenting with severe acute heart failure (HF), the endomyocardial biopsy (EMB) can be required to better understand underlying tissue abnormalities. Although it is currently performed in many tertiary centers, EMB remains an invasive procedure with potential, although rare, complications [3]. Cardiac magnetic resonance (CMR) can offer accurate cardiac volumetric and function assessments, as well as non-invasive and radiation-free myocardial tissue characterization [4]. However, its use in the acute setting has historically posed technical challenges due to the long imaging acquisition times and the need for appropriate breath-holding from a collaborative patient. CMR in acutely ill patients also poses operational challenges on how to safely manage this patient in an MRI environment, including deciding which patients are adequate candidates for the test and who are not. Some of the technical challenges can now be overcome by novel CMR sequences and focused imaging protocols, which significantly shorten the exam duration and increase the feasibility of the modality also in this setting.

## 2. Feasibility of CMR in Acutely Ill Patients

When CMR imaging is likely to impact clinical decisions, the process begins with evaluating the patient’s suitability for the procedure.

Ventilated patients may undergo CMR under certain conditions using an MRI-compatible ventilator; however, this is generally avoided unless there is a strong clinical indication [5]. The second step is to assess patients’ compliance and collaboration in maintaining a supine position for 20–45 min and the ability to follow breath-hold instructions. Difficulty in breath-holding and highly irregular heart rhythms can significantly degrade image quality and, consequently, reduce the diagnostic yield of the test [6].

## 3. Tailored CMR Protocol in Acute Cardiovascular Care

As previously mentioned, the first step to ensure the feasibility and good diagnostic yield of a CMR scan in the acute setting is a thorough discussion of clinical indications with the referring physician to tailor the acquisition protocol, avoiding unnecessary sequences.

A rapid and essential protocol, including mainly cine and late gadolinium enhancement (LGE) sequences, can be obtained in approximately 20 min [7]. Even when adding additional tissue characterization sequences and stress perfusion imaging, the scan time can be maintained below 35 min [8,9].

Every CMR protocol starts with a set of black-blood or bright-blood localizers that provide an initial anatomic assessment and allow for the planning of subsequent sequences [6]. Long- and short-axis cine images are then required for the volumetric and functional assessment; the latter are usually acquired during the required waiting time between gadolinium-based contrast agent (GBCA) injection and LGE sequence acquisition [6,7]. If an acute myocardial injury is suspected, T2-weighted images should be acquired to unveil myocardial edema, with the option to add parametric T2 mapping for quantification [6]. Although LGE remains the most robust parameter to non-invasively identify the presence and extent of myocardial fibrosis/necrosis, native T1 parametric mapping is a promising technique to detect diffuse or localized myocardial fibrosis without the need for a contrast agent [10]. An extracellular volume (ECV) estimation (interstitium and extracellular matrix) can be obtained using T1-mapping values before and after contrast administration and the patient’s hematocrit, either collected within 24 h [11,12] or generated using a synthetic, automated, ECV tool [13]. A pre-contrast T2* image allows for the identification of cardiac iron overload, a potential cause of HF and major arrhythmias in patients with thalassemia or hemochromatosis [12]. CMR has a lower temporal resolution than echocardiography [14]; however, both in-plane and through-plane phase contrast imaging can be acquired to visualize and quantify valve disease and intracardiac shunts [15]. Early gadolinium enhancement (EGE) images (within 3 min after contrast injection) can identify intracardiac thrombi and early microvascular obstruction (MVO), which may complicate acute myocardial infarction (MI) [6]. Perfusion sequences acquired at peak stress during either a vasodilator (i.e., adenosine, regadenoson, or dipyridamole) or an inotropic agent (i.e., dobutamine) injection can detect inducible myocardial perfusion defects; these appear as hypointense areas after GBCA injection compared to the normally perfused myocardium [16].

Shortening scan times can be achieved by reducing breath-holding times and/or the number of images acquired, using modern sequences accelerated by the implementation of artificial intelligence and machine learning technology. The breath-hold time should be maintained as short as possible and preferably below ten seconds to avoid breathing artifacts. Respiratory navigation can be used to remove the need for breath-holding. Real-time free-breathing images can be used instead of conventional breath-hold cine sequences to assess volumes and functions [6] in patients with arrhythmias and limited ability to breath-hold. Parallel imaging and artificial intelligence can further accelerate image acquisition [17]. Single-shot LGE sequences acquired during a single breath-hold allow for the rapid acquisition of multiple slices covering the whole heart [18]. In patients with cardiac implantable electronic devices (CIEDs), raising the arm can distance the device from the heart and mitigate the related artifacts. Wideband LGE sequences and spoiled gradient echo cine sequences, which are less sensitive to magnetic field inhomogeneities, can replace the standard sequences to further reduce device-related artifacts [19,20].

## 4. Clinical Scenarios

### 4.1. Acute Coronary Syndromes

**Figure 1:**
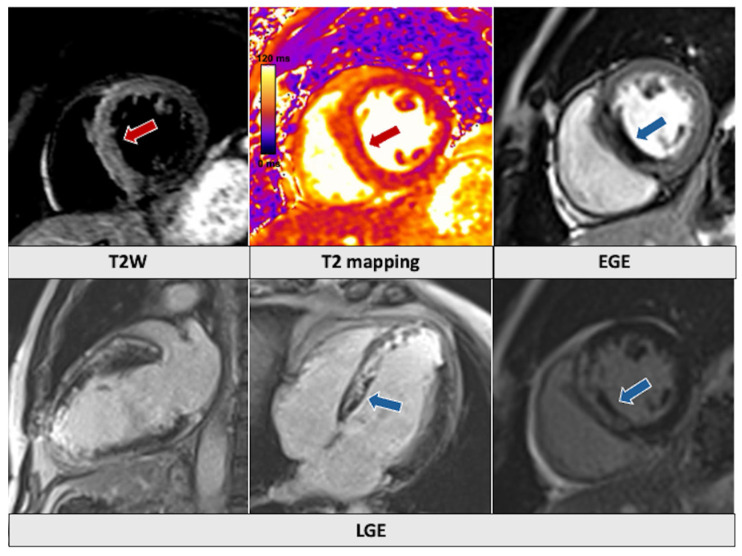
Patient admitted with anterior ST-elevation myocardial infarction. Cardiac magnetic resonance images showed evidence of increased signal intensity in T2-weighted images and increased T2-mapping values in the interventricular septum and anterior wall (red arrows). There is also evidence of microvascular obstruction in both early and late gadolinium enhancement images (blue arrows,) along with transmural LGE in the septum and apical segments. LGE: late gadolinium enhancement.

Acute coronary syndromes (ACSs) can be complicated by life-threatening arrhythmias or HF and represent the most prevalent cardiovascular disease requiring ICU admission [21]. Acute ischemic myocardial injury is detected at CMR imaging as an increased signal intensity on both T2-weighted sequences, indicating myocardial edema, and LGE sequences, where it has a subendocardial or transmural distribution in one or more coronary artery territories [22] (Table 1). The LGE extent is inversely correlated with a favorable prognosis, and dysfunctional segments characterized by LGE involving more than 50% of the myocardial thickness are unlikely to recover even after successful revascularization [22,23]. Thus, the evaluation of myocardial viability can be useful in guiding revascularization intervention, particularly when dealing with complex procedures and high-risk patients. Up to 50% of patients presenting with ST-elevation MI (STEMI) show MVO, a hypointense area within the myocardial scar in LGE images that indicates a persistent perfusion defect after revascularization [24] (Figure 1). In up to 40% of STEMI patients, there can also be evidence of intramyocardial hemorrhage (IMH) due to reperfusion injury within the necrotic myocardium, which appears as a hypointense core within a hyperintense area of myocardial injury in T2-weighted images [25]. Both MVO and IMH are markers of adverse remodeling and have been associated with an increased risk of death and cardiovascular events at follow-up [26].

CMR is the gold standard technique for identifying intracardiac thrombi, particularly when using EGE sequences with a long inversion time [25]. The thrombus is avascular, thus not enhancing in the signal after contract injection and appearing hypointense (dark), whilst the ventricular cavities are bright due to the presence of contrast.

CMR’s spatial and contrast resolution is also extremely helpful for the better characterization of ventricular aneurysms or pseudoaneurysms and to detect even small cardiac ruptures [27].

Pericardial inflammation, which can complicate MI even in the absence of pericardial effusion, appears as signal hyperintensity of the pericardial layers in both T2-weighted and LGE images (i.e., acute phase) or only LGE (i.e., subacute phase) [28]. 

In patients with multivessel disease, identifying the culprit lesion and distinguishing between acute and chronic events can be challenging; acute myocardial injury can be detected by CMR as areas of increased signal intensity on T2-weighted imaging [29]. In the absence of concomitant LGE, these areas are usually referred to as “areas at risk”, indicating they are injured but not infarcted and thus potentially viable in the case of reperfusion [30].

Up to 15% of patients presenting with ACS have MI with non-obstructed coronary arteries (MINOCA) at the coronary angiography [28]. Although the prognosis of these patients is usually good, they can experience some serious complications, including acute HF and cardiogenic shock [31]. The MINOCA diagnosis encompasses a heterogeneous group of patients with acute ischemic or nonischemic myocardial injury [31]. CMR is recommended by recent guidelines in class I in all patients with suspected MINOCA after a non-diagnostic invasive coronary angiography, to provide a final diagnosis [32]. The highest diagnostic yield is obtained when CMR is performed within two weeks from the acute event; this is particularly true for patients with troponin ≥211 ng/L, in which an early CMR was demonstrated to have a very high diagnostic yield of 94% [33]. Nevertheless, the diagnostic yield remains high at 72% even after 14 days from presentation. Results from the largest registry on MINOCA demonstrated that CMR reclassifies almost 80% of MINOCA diagnoses at the conventional assessment, thus guiding therapeutic management [34].

### 4.2. Takotsubo Syndrome

The Takotsubo syndrome (TTS) is a transient regional myocardial dysfunction typically involving mid-to-apical segments triggered by an emotional or physical stress. Atypical forms with focal or basal-to-mid segment involvement have also been described, with the latter representing up to 28% of all TTSs according to recently published data [35,36]. The clinical presentation can mimic ACS and can be complicated by life-threatening arrhythmias or cardiogenic shock, which is reported in up to 14% of patients [36]. Echocardiography can help to suspect the diagnosis, and invasive coronary angiography is required to exclude significant CAD [37]. However, diagnosing atypical and focal forms of TTS can be challenging at the conventional assessment. CMR can help in these cases by showing increased signal intensity in T2-weighted images in the affected segments in the absence of LGE [38,39]. The edema typically resolves within a few months, and it is accompanied by the resolution of regional wall motion abnormalities.

### 4.3. Pericardial Diseases

Echocardiography is the first-line tool for the initial evaluation of the pericardium [40]. However, this modality cannot assess the presence of inflammation [41], which is instead identified at CMR as a high signal intensity of pericardial layers in both T2-weighted and LGE images, particularly when using fat-suppression techniques [42]. A pericardial thickness greater than 4 mm and the evidence of ventricular uncoupling in real-time cine images acquired during free breathing suggest constrictive pericarditis [42]. This condition can also be present with a normal-thickness pericardium [43]. Cardiac tamponade is usually diagnosed by combining clinical and echocardiographic features [40]. However, rare, localized forms of cardiac tamponades can be missed and detected only at the CMR evaluation [42].

### 4.4. Myocarditis

Myocarditis is an inflammatory heart disease that can be secondary to immunological reactions, infections, or exposure to toxins [44]. Fulminant myocarditis is a severe and life-threatening form of myocarditis characterized by the sudden and rapid onset of myocardial inflammation, resulting in acute HF, cardiogenic shock, and life-threatening arrhythmias and requiring prompt diagnosis and treatment, often involving mechanical circulatory support [45,46,47]. The prognosis is particularly poor in the case of giant cell myocarditis, a form of necrotizing myocarditis characterized by rapid symptom progression and an 85% rate of death or heart transplantation at 3 years [44]. After the acute event, myocardial damage can eventually result in extensive adverse remodeling and dilated or non-dilated LV cardiomyopathy [48]. EMB is the gold standard for diagnosing myocarditis and its underlying etiology, particularly when presenting with hemodynamic instability; CMR is the main non-invasive modality for diagnostic and prognostic assessments and may guide EMB procedures by reducing sampling errors [49]. According to the updated Lake Louise criteria [50] acute myocardial inflammation can be confirmed with good accuracy with a combination of the positivity of at least one T2-based (T2-weighted or T2 mapping) and one T1-based criterion (T1 mapping, ECV, or presence of nonischemic LGE). The LGE pattern in these patients usually has a midwall or subepicardial, patchy distribution [51]. Larger areas of LGE, particularly with septal involvement, are associated with a higher risk of major cardiovascular events at follow-up [51].

### 4.5. Sarcoidosis

Sarcoidosis is an inflammatory granulomatous disorder mainly affecting lungs and lymph nodes but also other organs, including the heart, in isolated forms in 20–25% of patients [52]. The clinical presentation is highly variable, ranging from asymptomatic to life-threatening conditions, such as malignant ventricular arrhythmias, atrioventricular blocks, or HF [52]. Early detection is crucial for effective management. Both the Heart Rhythm Society and the European Association of Cardiovascular Imaging recommend Fluorodeoxyglucose Positron Emission Tomography (FDG-PET) and CMR as key diagnostic tools [53]. EMB alone has limited utility due to the focal nature of the disease [54], with a sensitivity estimated at 20–30% when not guided by imaging. However, combining FDG-PET with CMR, particularly with LGE, can help guide EMB, monitor treatment efficacy [55], and assess the risk of malignant ventricular arrhythmias [56]. LGE is typically located in the mid-wall or sub-epicardial regions of the basal lateral and septal walls but can have also subendocardial and transmural distributions. A meta-analysis of 10 studies and 760 patients demonstrated that patients with cardiac sarcoidosis and LGE had increased odds of both all-cause mortality and arrhythmogenic events at follow-up [56]. CMR can also identify inflammation via T2-weighted imaging, offering information about right ventricular involvement, with both associated with worse clinical outcomes [57].

### 4.6. COVID-19 Infection

Cardiac injury has been observed in 7–40% of COVID-19 patients [58], and its presence is linked to poor outcomes. Various mechanisms of cardiac injury have been identified in this context, such as active myocarditis mainly resulting from direct viral damage or a cytokine storm, stress-related cardiomyopathy, endothelialitis, and myocardial ischemia [58]. A prospective multicenter study found that the incidence of myocarditis in COVID-19 patients with elevated cardiac troponin was relatively low at 6.7% [59], while myocardial infarctions (particularly microinfarctions) represented the most common cause of cardiac injury; the presence of LGE was also a strong predictor of poor cardiovascular outcomes during follow-up [59]. Pericardial inflammation, with or without effusion, is another common cause of chest pain and hospital admission in COVID-19 patients, with similar diagnostic criteria and clinical management of other viral pericarditis [58].

### 4.7. Chagas Disease

Chagas disease secondary to Trypanosoma Cruzi infection is the leading cause of HF-related death in Central and South America [60]. Cardiac involvement may present as biventricular HF, remodeling, apical aneurysms, and arrhythmic events. LGE has been described in 16–69% of patients with Chagas disease, depending on the stage of the disease, and is more frequently located at apical and inferolateral segments [61]. The pattern is mainly transmural and subendocardial but also mid-wall and subepicardial, and the LGE extent is associated with the degree of LV dysfunction and the occurrence of ventricular arrhythmias [62]. Similarly, the presence of myocardial edema unveiled in T2-weighted CMR images correlates with the degree of ventricular dysfunction and identifies patients at a higher risk of developing a more severe form of the disease [63].

### 4.8. Heart Transplant Rejection

Despite the advances in clinical and therapeutic management, survival after heart transplantation remains significantly hampered by the risk of acute and/or chronic rejection, one of the main causes of morbidity and mortality along with coronary allograft vasculopathy (CAV). Early allograft failure is the principal cause of death during the first weeks after surgery and it manifests as severe biventricular dysfunction [64]. Acute rejection affects up to 40% of patients in the first year post-transplantation with presentation ranging from no symptoms to acute HF [65]. The gold standard for diagnosing graft rejection is EMB but, due to the focal nature of the disease, it could be a false negative in up to 20% of patients [66]. CMR can guide EMB, and a multiparametric approach, including T1 and T2 mapping, has shown high negative predictive value for graft rejection, resulting in a significant reduction in EMB referrals [67]. A recent metanalysis demonstrated that increased T2-mapping values are the single most reliable CMR predictor of rejection [68]. In contrast, LGE has lower specificity and performs poorly in this diagnostic setting [68]. Stress perfusion CMR is safe in post-transplantation patients and can help unveil perfusion defects associated with CAV [69].

### 4.9. Hypertrophic Cardiomyopathy and Phenocopies

**Figure 2:**
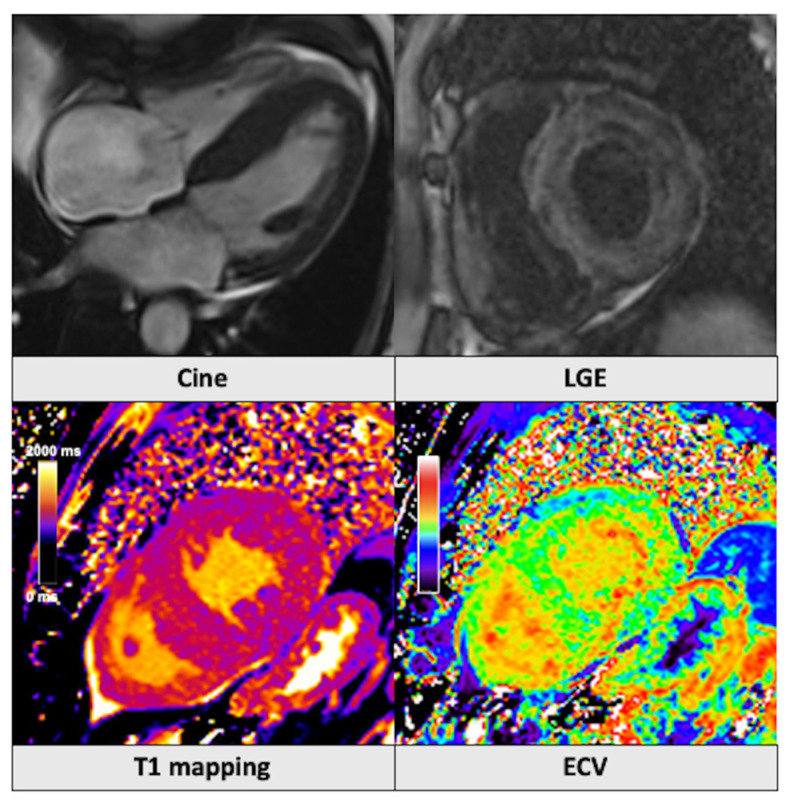
Patient admitted with worsening shortness of breath and leg swelling. Cine images demonstrated severe left ventricular hypertrophy with a maximum wall thickness of 22 mm. The typical LGE appearance with difficulty in nulling the myocardium along with significantly increased native T1-mapping (1176 ms) and ECV (48%) values were in keeping with the diagnosis of cardiac amyloidosis. ECV: extracellular volume. LGE: late gadolinium enhancement.

**Figure 3:**
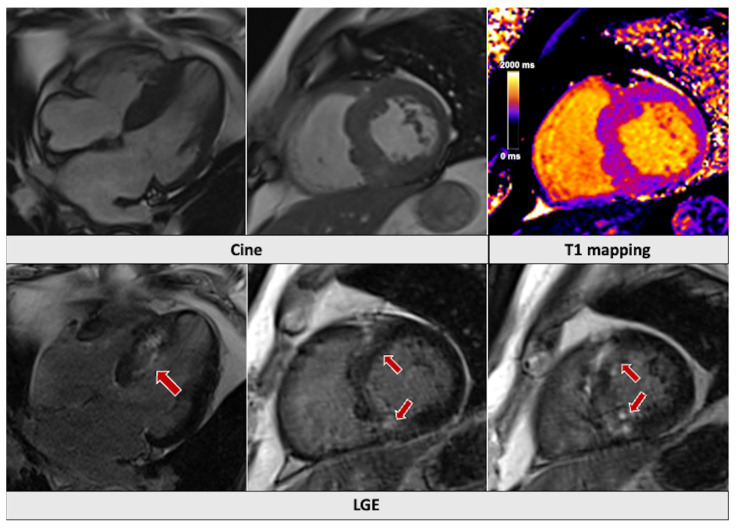
Patient admitted with shortness of breath and sustained ventricular tachycardia. There was evidence of severe, asymmetrical left ventricular hypertrophy, increased T1-mapping values (1086 ms), and patchy LGE involving both superior and inferior insertion points in keeping with the diagnosis of hypertrophic cardiomyopathy (red arrows). LGE: late gadolinium enhancement.

Malignant ventricular arrythmias and acute HF are potential life-threatening complications of hypertrophic cardiomyopathy (HCM) [70]. CMR is recommended for the initial evaluation and risk stratification of patients with suspected cardiomyopathies [70,71]. In fact, CMR is more accurate than echocardiography in assessing LV wall thickness and quantifying the LV mass [72], as well as in detecting LV apical aneurysms that complicate apical HCM, with a sensitivity of 97% compared to 67% for non-contrast echocardiography [73]. LGE involving ≥15% of the LV mass is an established major risk factor for SCD [72] and, among imaging variables, has demonstrated the highest prognostic value for SCD, all-cause mortality, and cardiovascular mortality in HCM patients [74]. Emerging evidence suggests that T1 mapping, alone or in combination with LGE, can also predict adverse cardiac outcomes [10,75,76]. Right ventricular dysfunction is also an emerging independent risk marker in HCM, whereas sole right ventricular hypertrophy is not [77]. CMR tissue characterization can aid in the differential diagnosis of hypertrophic phenocopies (Figure 2 and Figure 3).

This is important given that new specific disease-modifying therapies have shown the potential to improve both the quality of life and prognosis of these patients [78,79]. In Anderson Fabry disease, there are characteristic low T1-mapping values in the septum and mid-wall LGE in the basal inferolateral segment [80]. Circumferential subendocardial or transmural LGE patterns associated with difficulties in nulling the myocardium and markedly elevated T1 mapping and ECV values are suggestive of cardiac involvement in amyloidosis [81]. Finally, reduced T1-mapping values and T2* relaxation times are indicative of cardiac iron overload [82].

### 4.10. Dilated Cardiomyopathy and Phenocopies

Dilated cardiomyopathy (DCM) includes several conditions characterized by the impairment of LV systolic function associated with progressive LV dilatation [83]. These conditions can potentially evolve to end-stage HF and predispose the individual to malignant arrhythmias [84]. In this scenario, CMR can differentiate ischemic from nonischemic diseases, providing hints about the underlying genetic abnormalities [70]. The presence of subepicardial LGE with a “ring like” pattern (LGE involving of at least three contiguous segments in the same short-axis slice) is in fact more frequently associated with desmoplakin (*DSP*) and filamin C (*FLNC*) genes mutations and has a higher risk of heart transplantation, SCD, and major ventricular arrhythmias [84,85]. Nevertheless, about one third of DCM patients have LGE with a nonischemic distribution involving mainly the septum [85]. This finding is a predictor of adverse events at follow-up, including all-cause mortality and major arrhythmic events [86]. Interestingly, the association between LGE and SCD also remains significant in patients with an LVEF above the threshold recommended by the guidelines for ICD implantation (>35%) [86]. A combination of LGE with native T1 mapping and ECV has been recently demonstrated to be superior to only LGE and LVEF to predict the SCD risk [87]. The importance of LGE has also been recently demonstrated in the newly defined non-dilated LV cardiomyopathy phenotype; the presence of midwall LGE is in fact a significant predictor of MAACE in this highly heterogenous group of patients, aiding in risk stratification [88].

### 4.11. Cancer-Therapy-Related Cardiovascular Toxicity

Oncologic therapies are a well-known cause of cardiovascular toxicity [89]. The definition of “cancer-therapy related cardiovascular toxicity (CTRCT)” encompasses a broad spectrum of cardiovascular manifestations, such as new-onset HF, ventricular arrhythmias, or myocarditis [89]. Immune-check-point-inhibitor (ICI)-related myocarditis, described in about 1% of patients treated with these drugs, is a rare but serious CV complication that can present as cardiogenic shock with associated life-threatening arrhythmias [90]. In the uncomplicated case, this can be diagnosed using the CMR criteria described for other forms of myocarditis [90]. LGE with a nonischemic distribution has been described in up to 77% of patients with ICI-related myocarditis even in the presence of a normal LVEF and has been associated with worse CV outcomes at follow-up [90]. Cases of TTS have been reported with certain cancer therapies, including 5-fluorouracil, rituximab, and capecitabine [91]. Furthermore, a history of malignancy is an established risk factor for TTS. To identify CTRCT early, a serial imaging assessment is recommended as part of the clinical follow-up; in this regard, the same imaging modality should be preferred during follow-up, opting for radiation-free modalities [89].

### 4.12. Cardiac Masses

Cardiac masses are a rare but clinically relevant cause of HF and malignant ventricular arrhythmias due to mechanical obstruction, impaired valvular function, and cardiac infiltration. Cardiac metastases are more frequent than malignant primary cardiac tumors with pleural mesothelioma, melanoma, and lung adenocarcinoma representing the most frequent remote primary tumors [92]. Among primary cardiac tumors, fibromas and myxomas are the most frequent ones [93,94].

Sarcomas are the most common malignant primary cardiac tumor, followed by lymphomas and mesotheliomas [95]. They usually appear as isointense lesions on T1-weighted images, with nodular areas of higher intensity in cases of angiosarcomas (“cauliflower appearance”) [96]. The high cellularity and heterogeneous vascularization of malignant tumors usually result in the non-homogeneous enhancement at LGE sequences due to a necrotic core and T2 hyperintensity [96]. Right heart localization, local invasion, irregular borders, sessile attachment, associated pericardial effusion, or multiple lesions involving different cardiac chambers are all imaging red flags for malignancy [96]. Given the limited temporal resolution, CMR is of little help in characterizing small and mobile masses, such as vegetations and fibroelastomas, for which echocardiography remains the reference imaging modality [97].

## 5. CMR in Guiding Interventions in Critically Ill Patients

CMR can guide electrophysiological procedures by detecting underlying causes of heart block and malignant arrhythmias, stratifying the risk and optimizing procedural outcomes [98].

### 5.1. Tachycardia Ablation Procedures

In acute scenarios, ablation procedures may be required to treat arrhythmic storms that are unresponsive to medical therapy. By identifying the location and depth of arrhythmic substrates [99], CMR can aid in ablation procedures and reduce their duration and complexity, while increasing the likelihood of success. Both core scars (i.e., dense LGE areas) and border-zone scars (i.e., the interface between normal myocardium and core scar) have been identified as potential triggers of VT [100]. This can be distinguished by CMR exploiting differences in the signal intensity on CMR imaging and used to reconstruct colored 3D models to integrate with electroanatomical mapping [101]. Border-zone channels, recently emerging as VT substrates, can be identified as corridors of the normal myocardium connecting two areas of the normal myocardium located between two core areas or a core area and a valve annulus [101]. CMR-guided-only procedures, without any use of electoanatomical mapping, have also been tested in the research setting and demonstrated potential in reducing procedural, fluoroscopy, and radiofrequency times [99].

However, LGE imaging may have limitations in the acute setting, as it may overestimate the myocardial infarct size due to the concomitant presence of myocardial edema (also bright in LGE sequences) in the peri-infarct region. Additionally, LGE-CMR presents limitations to detect subtle or diffuse pathological processes [102]. The implementation of T1-mapping techniques can help to address these limitations; despite being supported by less evidence, parametric mapping has been used in the research setting for this purpose [103].

### 5.2. CIED

In patients presenting with advanced AV blocks, CMR can investigate the presence of underlying cardiomyopathies [70]. LGE quantification is also key to improving arrhythmic risk stratification [104]. A primary prevention implantable cardioverter defibrillator (ICD) is the key therapy for SCD prevention and is currently recommended based on two main parameters: New York Heart Association class and LVEF <35% [105]. However, current risk-stratification strategies proved to be not sufficient to accurately identify patients at a higher risk of SCD, as only 1 in 5 patients receive appropriate ICD shocks over 5 years in major HF trials [106]. The presence and extent of LGE was demonstrated to predict arrhythmic outcomes in several clinical settings, both ischemic and non-ischemic [107,108]. The most recent European guidelines on cardiomyopathies therefore recommend to also consider primary-prevention ICD implantation in patients with LVEF >35% when additional risk factors are present, including significant LGE in the presence (class IIa, level of evidence C) or absence of high-risk genotype (class IIb, level of evidence C) [81]. An LGE evaluation may also be useful to guide resynchronization therapy; the absence of LGE in the inferolateral wall predicts a good clinical response to cardiac resynchronization therapy (CRT) [109], while pacing over LV inferolateral scars, particularly if large and transmural, is associated with a poor response and increased arrhythmic risk [110]. CMR can, therefore, identify patients who may benefit most from CRT implantation and may have a role in guiding LV lead deployment away from the scarred myocardium [110].

## 6. Other Potential CMR Applications in Critically Ill Patients

### 6.1. Acute Aortic Syndromes

Acute aortic syndrome (AAS) refers to a group of life-threatening aortic diseases characterized by sudden-onset chest or back pain, potentially leading to severe complications if not promptly diagnosed and treated. This includes aortic dissection, intramural hematoma, and penetrating atheromatous ulcers, with CT being the first-choice imaging modality in cases of AAS suspicion. CMR has longer acquisition times and lower spatial resolution and is not widely used in clinical practice for this purpose unless CT is contraindicated or a radiation-free modality is preferred as part of the serial follow-up assessment. In the case of aortic dissection, CMR can visualize and quantify flows in both the true and false lumen, with the latter typically having a larger diameter and a slower flow [111]. An aortic intramural hematoma (AIH) can appear as a thickened aortic wall with signal characteristics typically related to hemoglobin-degradation products; the signal is usually isointense on T1-weighted and hyperintense on T2-weighted images in the acute phase [112]; the signal then becomes hyperintense in both T1- and T2- weighted images in the subacute phase due to the change from oxyhemoglobin to methemoglobin [111]. A mural thrombus, which is often difficult to distinguish from AIH, appears instead as hypointense or isointense in both T1-weighted and T2-weighted sequences [111]. Penetrating aortic ulcers occur when atherosclerotic plaques disrupt the intima, appearing as a contrast-filled outpouching of the lumen that can evolve into AIH, aortic dissection, or aortic rupture. Aortitis, which may be misdiagnosed as AIH, appears as a homogeneous circumferential thickening of the aortic wall, hyperintense in T2-weighted imaging [113].

### 6.2. Cardiac Traumas

Blunt cardiac injury that can occur during chest traumas is a rare cause of malignant arrhythmias and cardiac rupture. The clinical management of these patients is challenging due to the absence of a gold-standard diagnostic test. A recent pilot trial enrolling 42 patients with suspected blunt cardiac injury showed that sensitivity and specificity values for CMR in predicting MACE were 60% and 81%, respectively. [114].

### 6.3. Valvular Heart Disease

Echocardiography is the first imaging modality used to assess the presence and degree of valvular heart disease. CMR can provide the accurate quantification of regurgitations [115,116], often challenging at the multiparametric echocardiographic evaluation, and for the risk stratification of malignant ventricular arrhythmias in patients with mitral valve prolapse [117]. In the latter condition, CMR can comprehensively evaluate the risk factors associated with arrhythmias, including mitral annulus disjunction and LGE [118,119]. Due to longer CMR acquisition times along with the need for patient compliance, echocardiography remains the preferred modality to evaluate valvular disease in the acute setting, both for its faster execution and for its wider availability [1].

## 7. Limitations and Contraindications

A careful clinical evaluation of critically ill patients is essential before proceeding with CMR; the limited cooperation with breath-holding or the inability to remain still can severely compromise the image quality. Moreover, the relatively long duration of the exam may pose significant clinical risk for unstable patients. Personnel fully trained in advanced cardiovascular and respiratory support, as well as resuscitation equipment, should be readily available near the scanner room, with the mandatory continuous monitoring of vital parameters throughout the study. MRI facilities are commonly equipped with piped-in oxygen, and, when required, nasal administration is possible during the scan. MRI-safe infusion pumps also allow for continuous drug infusion in patients under intensive i.v. treatment. CMR is instead contraindicated in patients with non-MRI-conditional cardiac devices, including hemodynamic support devices (i.e., intra-aortic balloon pump; impella device; veno-arterial extracorporeal membrane oxygenation; left and right ventricular assist devices), MRI-conditional CIEDs implanted <6 weeks, and abandoned and epicardial leads.

The use of GBCA can occasionally cause allergic reactions [120] or, very rarely, lead to nephrogenic systemic fibrosis in patients with acutely impaired renal functions [121]. To mitigate these risks, GBCA should be used at the lowest effective dose in patients with significant renal impairment [122].

The cost-effectiveness of CMR in the acute setting may appear as a limitation, but the few available data suggest that the routine use of CMR in acute myocardial infarction with normal coronary angiography is associated with a reduction in costs in the medium to long term, due to a more personalized and higher quality of care over time that offsets the initial higher cost of the exam [123]. The availability of a dedicated CMR slot, along with the issue in ensuring safety in the transfer of critically ill patients to a CMR facility, particularly if requiring continuous monitoring or mechanical support, poses other significant challenges to the wider adoption of the modality.

## 8. Conclusions

**Figure 4:**
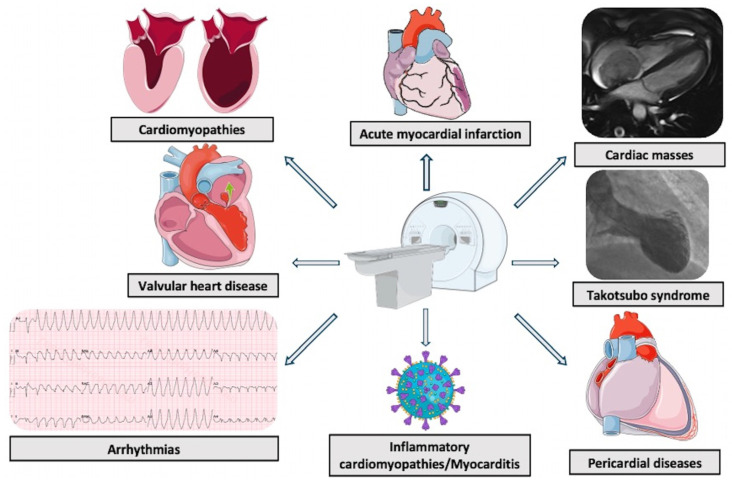
Cardiac magnetic resonance applications in acutely ill patients.

CMR has shown remarkable potential in diagnosing and managing patients in intensive and acute cardiovascular care settings. Its ability to deliver non-invasive, accurate myocardial tissue characterization, combined with volumetric and functional assessments, positions it as an essential tool for diagnosis, prognosis, and therapeutic decision-making across a range of acute cardiovascular conditions (Figure 4). While challenges related to patient stability, MRI compatibility of therapeutic equipment, and adequate image quality in the presence of arrhythmias or non-cooperative patients remain, advancements in technology and methodology are enhancing its feasibility and use even in the intensive and acute care setting.

## Figures and Tables

**Table 1:** Summary of main CMR role and findings.

Clinical Scenario	Main CMR Role and Findings
Acute coronary syndrome	Distinction between ischemic and non-ischemic causes of myocardial injury in patients with a MINOCA working diagnosis. Ischemic myocardial injury presents with increased signal intensity on both T2-weighted and LGE sequences, with a subendocardial or transmural pattern in one or more coronary artery territories, differently from non-ischemic injury, for which the pattern might be mid-wall, patchy, or subepicardial Micro-vascular obstruction: hypointense core within an ischemic injury on LGE sequences Intramyocardial hemorrhage: hypointense core within an ischemic injury on T2-weighted images Detection of post-myocardial infarction mechanical complications, including aneurysms, pseudoaneurysms, myocardial rupture, pericarditis, and thrombi. Thrombi present as endo-cavitary masses without enhancement on both early and late gadolinium enhancement sequences Distinction between acute ischemic injury (i.e., both T2-weighted and LGE-positive) and previous myocardial infarction (i.e., only LGE-positive)
Takotsubo syndrome	Hypokinesia/akinesia of the left ventricular apex associated with increased signal intensity on T2-weighted images typically without LGE in the typical forms
Myocarditis	Lake Louise diagnostic criteria based on at least one T2-based (increased signal on T2-weighted or increased values on T2 mapping) and one T1-based (increased values on native T1 mapping, increased ECV, or non-ischemic LGE) criterion
Pericardial diseases	Pericarditis High signal intensity of pericardial layers in both T2-weighted and LGE sequences (i.e., acute phase) or only LGE (i.e., subacute phase) Constrictive pericarditis Increased pericardial thickness (> 4 mm) Septal bounce on real-time cine imaging during deep inspiration/expiration
Hypertrophic cardiomyopathy	Gold standard for LV mass quantification LV apical aneurysms detection Tissue characterization using LGE and parametric mapping allows for differential diagnoses among hypertrophic phenocopies and risk stratification. LGE involving ≥15% of total LV mass is considered a risk marker for SCD
Dilated cardiomyopathy	Differential diagnosis between ischemic and non-ischemic causes according to LGE pattern Risk stratification according to LGE presence
Sarcoidosis	LGE can present with all patterns (i.e., subepicardial/midwall or transmural/subendocardial), more commonly located in the basal septal or lateral segments. Association with aneurysms, increased signals on T2-weighted sequences, or right ventricular involvement is a characteristic
Heart transplantation	Surveillance for rejection using T1-T2 mapping imaging, and conventional T2-weighted and LGE sequences Stress perfusion CMR might unveil cardiac allograft vasculopathy
Anderson-Fabry disease	Decreased native T1-mapping values as a sign of early cardiac involvement of the disease Mid-wall LGE in the basal inferolateral segment is a late finding and associated with the worst clinical outcomes and reduced likelihood of a response to enzyme replacement therapy
Cardiac amyloidosis	Difficulty in nulling myocardial signal on LGE sequences Circumferential subendocardial or transmural LGE pattern Markedly elevated native T1 mapping and ECV values
Cardiac iron overload	Reduced native T1-mapping and T2* relaxation times
Chagas disease	LGE with heterogenous patterns but typically located at apical and inferolateral LV segments, often associated with increased signal intensity on T2-weighted imaging
Valvular heart disease	Mitral valve prolapse Visualization and quantification of valve regurgitation using cine and phase-contrast images Visualization and quantification of concomitant MAD and associated LGE for SCD risk stratification
Cardiac masses	Malignant tumors usually present with infiltration, larger dimensions, and non-homogeneous post-contrast enhancement. They might be associated with pericardial/pleural effusions
Ventricular tachycardia ablation procedures	Identification of VT substrate using LGE imaging Identification of border zone channels as corridors connecting two areas of normal myocardium between two core LGE areas or a core LGE and a valve annulus
CIED	LGE assessment to aid risk stratification in patients who are candidates for ICD implantation Large, transmural scars located in the inferolateral walls predicts poor response to CRT or suggest alternative areas for pacing
Acute aortic syndromes	Alternative technique when CT cannot be performed, and TOE is inconclusive. It mainly allows for visualization of true and false lumens in aortic dissection, thickened aortic wall in intramural hematoma, and hyperintense signal of the aortic wall on T2-weighted imaging in aortitis

Legend: CMR, cardiac magnetic resonance; CRT, cardiac resynchronization therapy; ECV, extracellular volume; ICD, implantable cardioverter-defibrillator; LGE, late gadolinium enhancement; LV, left ventricle; MAD, mitral annular disjunction; MINOCA, myocardial infarction with non-obstructive coronary arteries; SCD, sudden cardiac death; TOE, transesophageal echocardiography; VT, ventricular tachycardia.

## Abbreviations

AAS	Acute aortic syndrome
ACS	Acute coronary syndrome
AIH	Aortic intramural hematoma
AV	Atrioventricular
CAD	Coronary artery disease
CT	Computed tomography
CIED	Cardiac implantable electronic devices
CMR	Cardiac Magnetic Resonance
CTRCT	Cancer-therapy-related cardiovascular toxicity
CRT	Cardiac resynchronization therapy
DCM	Dilated cardiomyopathy
ECV	Extracellular volume
EGE	Early gadolinium enhancement
EMB	Endomyocardial biopsy
FDG-PET	Fluorodeoxyglucose Positron Emission Tomography
GBCA	Gadolinium-based contrast agents
HCM	Hypertrophic cardiomyopathy
HF	Heart failure
ICD	Implantable cardioverter defibrillator
ICU	Intensive care unit
IMH	Intramyocardial hemorrhage
MACE	Major adverse cardiovascular event
MI	Myocardial infarction
MRI	Magnetic resonance imaging
LGE	Late gadolinium enhancement
LV	Left ventricle
LVEF	Left ventricular ejection fraction
MINOCA	Myocardial infarction with nonobstructive coronary arteries
MVO	Microvascular obstruction
STEMI	ST-elevation myocardial infarction
SCD	Sudden cardiac death
TTS	Takotsubo syndrome
